# Real-time analysis of methylalumoxane formation†

**DOI:** 10.1039/d0sc05075j

**Published:** 2020-10-27

**Authors:** Anuj Joshi, Harmen S. Zijlstra, Elena Liles, Carina Concepcion, Mikko Linnolahti, J. Scott McIndoe

**Affiliations:** Department of Chemistry, University of Victoria PO Box 1700 STN CSC, Victoria BC V8W 2Y2 Canada mcindoe@uvic.ca; Department of Chemistry, University of Eastern Finland P.O. Box 111 FI-80101 Joensuu Finland

## Abstract

Methylalumoxane (MAO), a perennially useful activator for olefin polymerization precatalysts, is famously intractable to structural elucidation, consisting as it does of a complex mixture of oligomers generated from hydrolysis of pyrophoric trimethylaluminum (TMA). Electrospray ionization mass spectrometry (ESI-MS) is capable of studying those oligomers that become charged during the activation process. We have exploited that ability to probe the synthesis of MAO in real time, starting less than a minute after the mixing of H_2_O and TMA and tracking the first half hour of reactivity. We find that the process does not involve an incremental build-up of oligomers; instead, oligomerization to species containing 12–15 aluminum atoms happens within a minute, with slower aggregation to higher molecular weight ions. The principal activated product of the benchtop synthesis is the same as that observed in industrial samples, namely [(MeAlO)_16_(Me_3_Al)_6_Me]^−^, and we have computationally located a new sheet structure for this ion 94 kJ mol^−1^ lower in Gibbs free energy than any previously calculated.

## Abstract 2

The activator methylaluminoxane is made by hydrolysis of trimethylaluminum. Analysis using ESI-MS reveals rapid formation of small oligomers is followed by slower aggregation to the larger precursors most capable of releasing [Me_2_Al]^+^.

## Graphical abstract

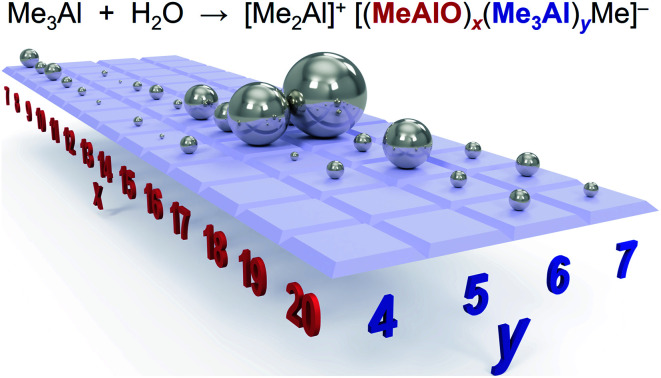

## Introduction

Methylalumoxane (MAO) is an oligomeric activator for single-site olefin polymerization precatalysts, prepared by the reaction of trimethylaluminum (TMA) with water.^1–7^ MAO is a complete activator^8–10^ through playing multiple roles: it acts as a scavenger of oxygen and water; it can alkylate the precatalyst; and it can ionize the precatalyst *via* abstraction of a methyl group.^11,12^ Trimethylaluminum is a capable scavenger on its own,^13^ and will also methylate metal–halogen bonds,^14,15^ but it is not able to ionize the precatalyst.^16^ MAO is however expensive due to the high aluminum to metal ratios required to achieve high productivities, with ratios of 10^4^ being typical.^8^ A limited understanding of the structure of MAO has hampered efforts to improve its efficiency. Different grades of MAO are available commercially containing varying amounts of unreacted TMA arising from incomplete hydrolysis.^11,17^ The TMA in MAO can be divided into two kinds: “bound TMA” which is incorporated in the MAO and “free TMA” which can be removed under vacuum to form TMA-depleted MAO (DMAO).^18^ Free TMA can be effectively trapped by adding a sterically hindered phenol such as 2,6-di-*tert*-butyl-4-methylphenol (BHT).^19,20^ The catalytic productivity and polymer molecular weight depends on the amount of free TMA in MAO and its synthesis history.^19,21–27^ Replacing the methyl group in MAO by bulkier alkyl groups such as isobutyl or octyl leads to the formation of modified MAO (MMAO), which have increased solubility and stability.^11,21,28,29^ Ionization in MAO comes about *via* neutral MAO generating the reactive Lewis acidic species [Me_2_Al]^+^, with the resulting bulky MAO anions being sufficiently weakly coordinating to allow high reactivity towards alkenes at the cationic metal center.^30–34^ We have shown through mass spectrometric means that the anionic products of the activation process are dominated by a single ion, [(MeAlO)_16_(Me_3_Al)_6_Me]^−^ henceforth [16,6]^−^.^35^ The three dimensional structure of this anion has not been elucidated, but its unusually high abundance in the spectra of post-activation commercial MAO does raise questions about why it is so prominent, since the synthesis of MAO does not on the face of it appear to be particularly selective, being the controlled mixing of water and pyrophoric TMA.^36^ Laboratory scale syntheses of hydrolytic MAO use hydrated salts^37,38^ to slowly release the water such that controlled hydrolysis of TMA is possible. Direct hydrolysis of TMA by the use of ice^39^ or wet solvent^40,41^ has also been reported. Alternative methods for preparation of MAO from reaction of benzoic acid, CO_2_ with TMA or from the reaction of TMA with Me_3_SnOH have been reported.^42–44^ The appearance of a “magic” ion that dominates a mixture with a broad distribution of possible products has always attracted attention from curious chemists. For example, time-of-flight mass spectra of laser-vaporized graphite reveals a range of (C_2_)_*n*_ ions, of which C_60_ was the most abundant component thanks to the special stability of the truncated icosahedral structure of that molecule.^45^ Protonated water droplets, [H(H_2_O)_*n*_]^+^, feature [H(H_2_O)_21_]^+^ as an especially prominent ion, thanks to the stability of a water molecule surrounded by 20 others in an icosahedral array.^46^ Understanding the special stability of [16,6]^−^ is challenging due to the pyrophoric nature of the matrix itself, so separation of this component is exceptionally challenging. As such, we resolved to discover what we could about the generation of this ion by real-time monitoring of the synthesis process itself, and to delve deeper computationally into its structure.

**Fig. 1:**
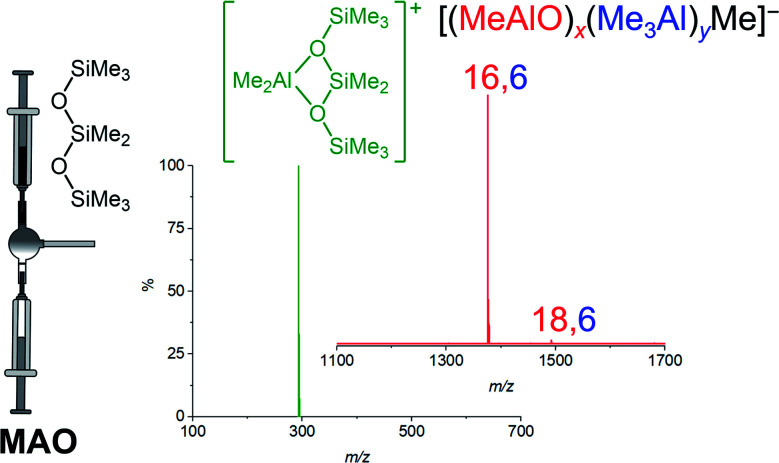
Ionization of MAO to generate [Me_2_Al(OMTS)]^+^ (green) and predominantly [16,6]^−^ with small amount of [18,6]^−^.

ESI-MS reveals predominantly [16,6]^−^ ion in MAO solutions in the presence of any additive that reacts readily with [Me_2_Al]^+^. Cp_2_ZrMe_2_ generates [Cp_2_ZrMe] [16,6]^−^, [NBu_4_]Cl generates [NBu_4_][16,6]^−^, but the most convenient way to make the ion is *via* addition of octamethyltrisiloxane (Me_3_SiOSiMe_2_OSiMe_3_, OMTS). OMTS chelates available [Me_2_Al]^+^ to generate [Me_2_Al(OMTS)]^+^ (Fig. 1).^35,47^ The resulting anion can be characterized in negative mode ESI-MS. We have used this technique to study alkyl exchange,^29^ aging,^48^ and oxidation^49^ of MAO, where the anion distribution changes in response to these processes. Here we report the dynamic behaviour of MAO anions formed *via* the reaction of TMA and water.

## Results and discussion

When water and TMA are combined, a fast exothermic reaction generates MAO with methane as a byproduct.^50^ We faced severe methodological challenges in studying this system mass spectrometrically, because of the evolution of methane, the exothermicity of the reaction, the low polarity of the toluene solvent^51^ generally used in synthesis, the propensity of the reacting solution to cause capillary blockages during analysis, the complexity of the mixture, and the inapplicability of normalization in the context of a system whose total ion count is changing. These factors conspired together to give extremely noisy time course data (see ESI†), though with consistent trends in speciation. ESI-MS analysis in fluorobenzene or difluorobenzene provided essentially the same collection of ions but with increasingly better ion intensity as the solvent polarity increased. Speciation was largely unaffected by whether OMTS was added at the start of the reaction or at the time of analysis (see ESI†).

**Fig. 2:**
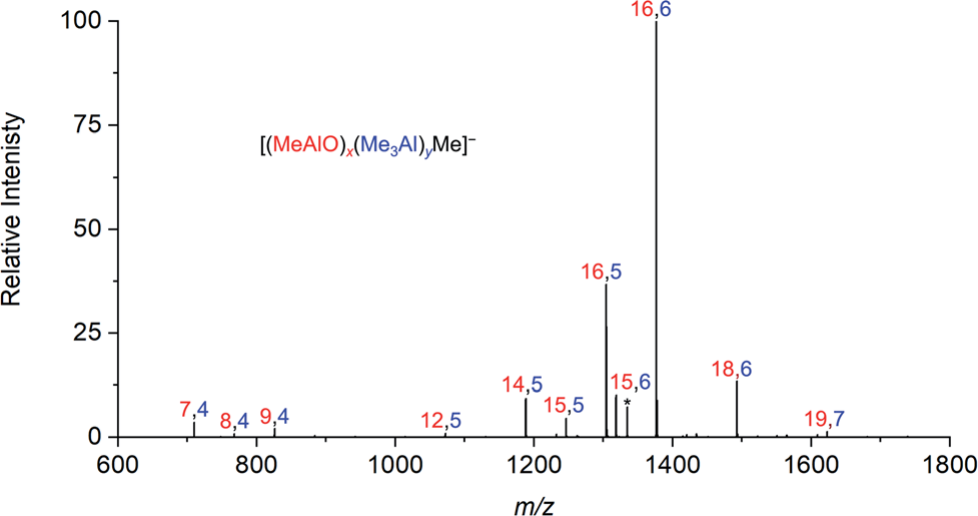
Summation of all negative ion ESI mass spectra collected for 30 minutes after mixing of TMA, wet (0.055 M H_2_O) degassed difluorobenzene, and OMTS.

**Fig. 3:**
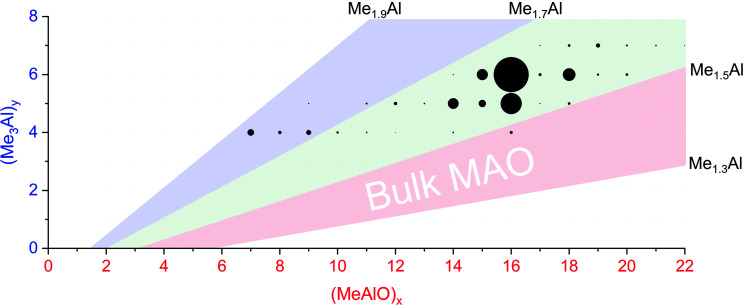
Plot of mass spectrometric intensities (proportional to circle area) from Fig. 2 against *x* and *y*. The pink area shows Me : Al ratios between 1.3 to 1.5, the proportions reported for bulk MAO. The green area shows Me : Al ratios between 1.5 to 1.7, observed for nearly all anions except for the lowest mass ions observed (blue, Me : Al 1.7 to 1.9).

The rate of reaction was significantly affected by the amount of water present, and the reaction could be slowed considerably by reducing the concentration of water used. The water concentration in the solvent was measured after the addition of Cp_2_ZrMe_2_ by ^1^H NMR.^52^ None of the reaction components (TMA, H_2_O, OMTS, difluorobenzene) on their own provide significant quantities of ions, but their combination generates alumoxane species capable of ionizing *via* capture of [Me_2_Al]^+^ by OMTS. More than 99% of the ion current during the hydrolysis experiments could be assigned to ions of the form [(MeAlO)_*x*_(Me_3_Al)_*y*_Me]^−^ (Fig. 2), hence the general formula (MeAlO)_*x*_(Me_3_Al)_*y*+1_ for the neutral precursors applies for those alumoxanes competent to act as activators. The empirical formula of bulk MAO has been established by NMR^53^ to fall in the range Me_1.3–1.5_AlO_0.75–0.85_. Nearly all the activator species we observe are comparatively rich in Me_3_Al (all of them having higher Me and lower O content, in the range Me_1.5–1.8_AlO_0.58–0.73_ (Fig. 3)).

**Fig. 4:**
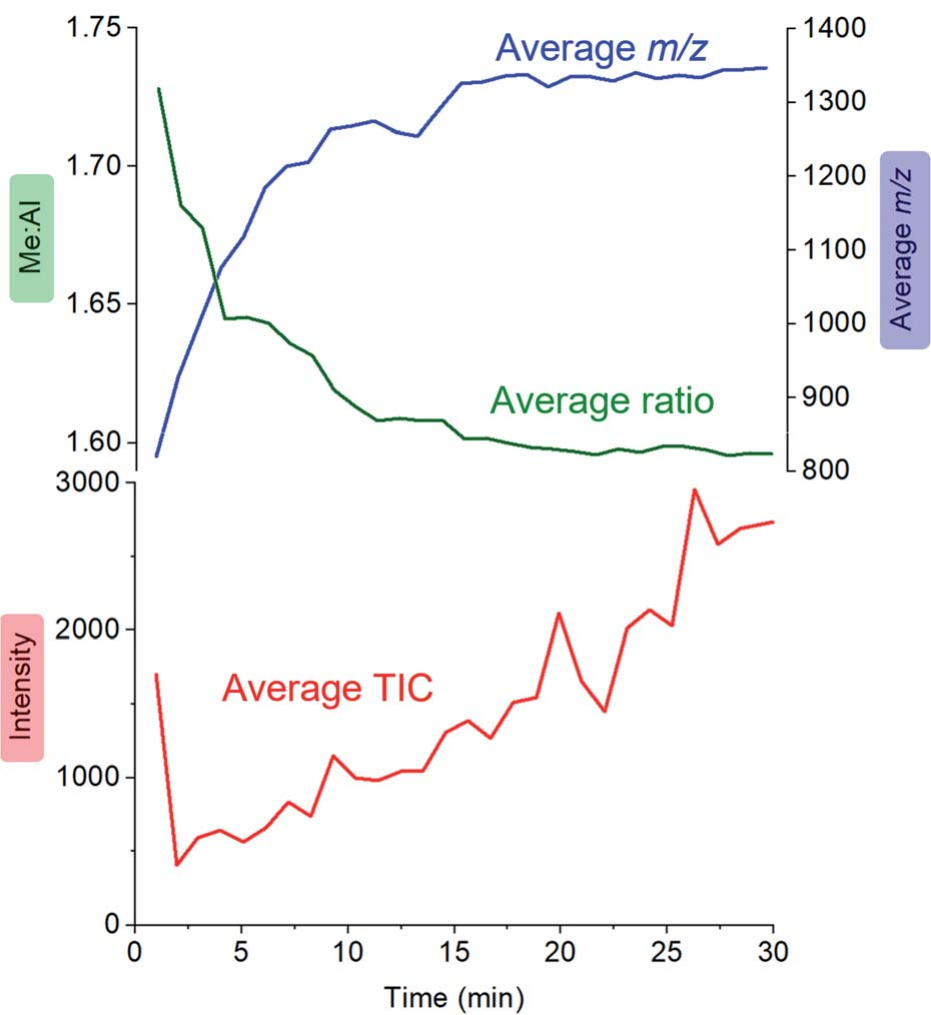
Plot of total ion current (TIC, red), Me : Al ratio (green) and average *m*/*z* (blue) as a function of time, for the reaction of TMA with water followed by ionization using OMTS.

Activator precursors have the empirical formula (MeAlO)_*n*_(Me_3_Al)_(0.36–0.71)*n*_, and are only observed when *n* > 6. The mass spectrometric results must be interpreted carefully because they encapsulate two separate processes: increase in molecular weight through oligomerization, and the propensity for species to ionize *via* [Me_2_Al]^+^ loss. As a result, the mass spectrometric abundance of a particular alumoxane is proportional to both its concentration and its extent of ionization (complicated further by the fact that not all ions have the same response even at the same concentration due to variations in surface activity,^54^ but given these ions are closely related these differences are likely to be comparatively minor). While the selectivity for ionized species complicates the analysis, it is nonetheless invaluable because it allows for molecular identification of *only* those species responsible for catalyst activation. We can extract three collective data sets out of a monitoring run: the total ion count, the average Me : Al ratio and the average mass-to-charge ratio (Fig. 4). The ion intensity is high when the reacting solution first reaches the mass spectrometer, but rapidly drops away, and subsequently climbs again slowly. The average *m*/*z* value starts at ∼800, climbs rapidly to ∼1300, and very slowly climbs to approximately *m*/*z* 1350. The average Me : Al ratio starts at ∼1.75 and drops to ∼1.6, slowly decreasing after that to ∼1.58.

MAO oligomers are produced extremely rapidly (in the few seconds before the reaction solution even reaches the mass spectrometer), commensurate with the high reactivity of TMA with water. The initial stages of reaction probably involve a cascade of hydrolysis, oligomerization, and isomerization reactions.^55–58^ Species of *m*/*z* +2 (ions with –OH in place of –Me) were observed only in trace amounts, suggesting that these components of the mixture are short-lived in solution. Computationally it is possible to predict the lowest energy structures for a given *x*,*y* combination,^59,60^ but the solution is evolving extremely quickly and we expect it to be a complex mixture of kinetic products, with linear, ring and ladder-type structures all present and prone to reaction with each other, any proximal –OH groups on other MAO oligomers, and with Me_3_Al.^61–64^

**Fig. 5:**
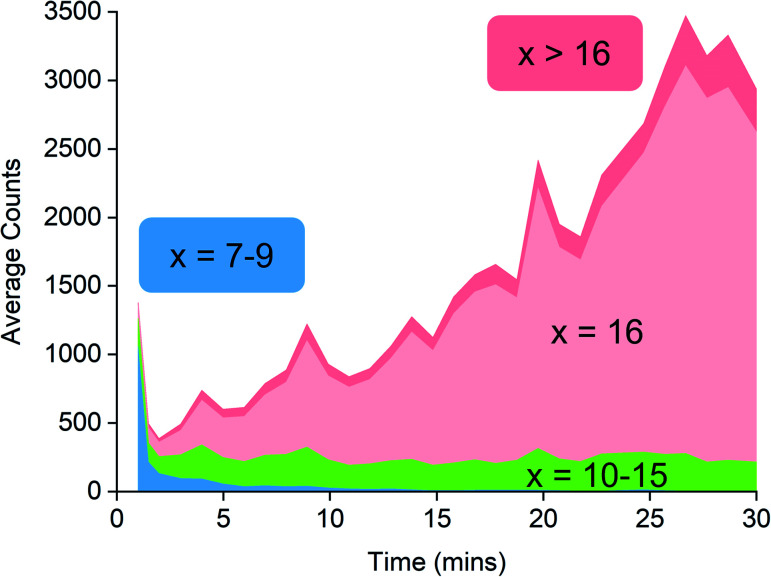
Ion intensity by *x* value, classified into different groups: blue (*x* = 7–9), green (*x* = 10–15), pink (*x* = 16) and red (*x* >16). *x* refers to the number of (MeAlO) units as the general formula for the anion is [(MeAlO)_*x*_(Me_3_Al)_*y*_Me]^−^.

Examination of the ions contributing to the total ion current provides a more complete picture. Early on in the reaction, the initial high intensity is produced almost entirely by three ions: [7,4]^−^, [8,4]^−^ and [9,4]^−^, suggesting that these ions are generated by the lowest mass precursors capable of acting as activators (Fig. 5). Previous computations indicate that sheet structures dominate in this size domain, and beginning from (MeAlO)_8_(Me_3_Al)_5_, *i.e.* the neutral precursor for [8,4]^−^*via* [Me_2_Al]^+^ loss, the sheets undergo transition from Al five-coordinate to Al four-coordinate structures.^30^ Slower reactions were also performed using lower concentrations of H_2_O, and these three ions were still the lowest mass ions observed (see ESI). The three ions have relatively high Me : Al ratios and are short lived, declining to baseline levels within a couple of minutes. Despite their effectiveness at ionization, they are unlikely to contribute to the performance of MAO, because their time in solution is so short-lived.

**Scheme 1:**
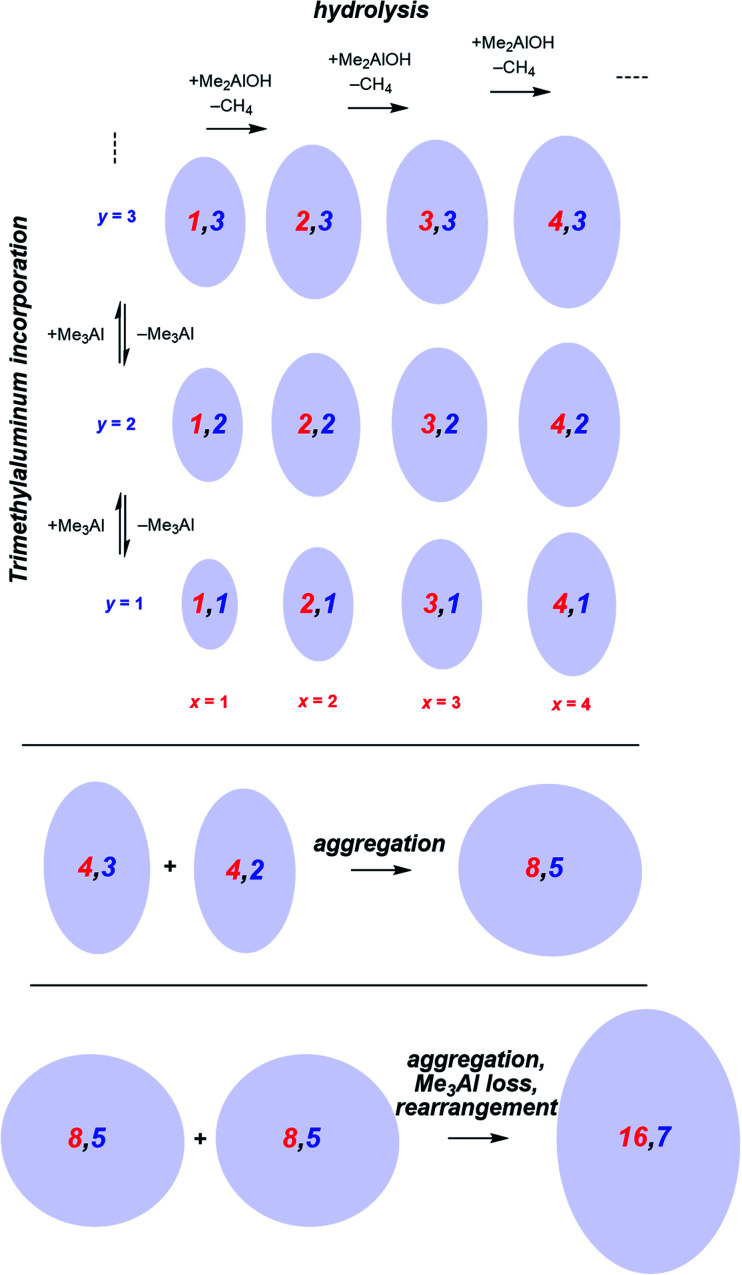
Plausible processes contributing to oligomerization: top, fast processes; bottom, slower aggregation. Structures shown are systematic examples; many isomers exist for each *x*,*y* combination.

Following the brief appearance of [*x*,4]^−^ (*x* = 7, 8, 9) the total ion current dips, and the three intense ions are not correspondingly replaced by incrementally larger oligomers. Instead, we see ions of much higher molecular weight, prominent amongst which is the “magic” [16,6]^−^ ion, whose abundance steadily climbs over the 30 minutes of reaction time. Of the many potential ions of intermediate composition, we see only a limited subset: small amounts of [11,4]^−^, [12,5]^−^, [14,5]^−^ and [15,5]^−^. At long reaction times, we observe [18,6]^−^ and [19,7]^−^, ions previously observed in aged MAO solutions.^48^ The very fast production of the [*x*,4]^−^ (*x* = 7, 8, 9) species and the gradual emergence of higher mass species suggests that the oligomerization process involves multiple processes with very different rates. Given the high reactivity of water and TMA, free water will not survive for an appreciable duration. The early stages of oligomerization are probably dominated by reactions involving methane loss (*i.e.* reactions between Al–OH and Al–Me) and incorporation of TMA. The slower production of higher molecular weight species is likely the result of aggregation of smaller neutral methylalumoxane fragments (Scheme 1).^65^ The progressive reduction in Me : Al ratio as the reaction proceeds points towards aggregation processes accompanied by loss of TMA. A possible explanation for the drop in ion current after the initial surge is due to aggregation processes forming open, high molecular weight, Me-rich structures that are ineffective activators until TMA attrition and subsequent rearrangement renders them capable of activation (ionization) through efficiently delocalizing the resulting negative charge.

Combining the experimental results to our ongoing computational studies on MAO using Gaussian 16 software^66^ with M06-2X^67^ DFT functional of the Minnesota series (as recommended for systems with dispersive interactions due to bridging Al–Me bonds)^26^ in combination with the def-TZVP basis set,^68^ allows us to propose a new structural model for the dominant [16,6]^−^ anion. The procedure for its location involves thorough screening of TMA hydrolysis^59^ and anionization reactions,^31^ and will be reported in detail elsewhere.

**Fig. 6:**
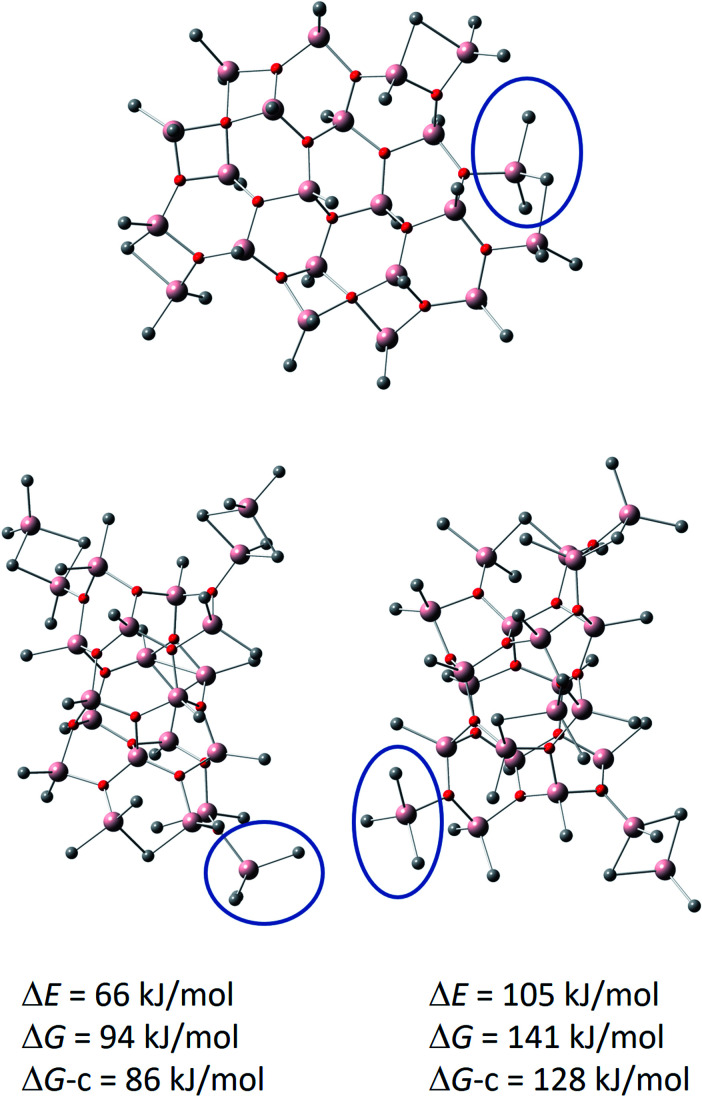
Calculated structure of [16,6]^−^ sheet (top) with comparisons to previously reported cage anions. Bottom left: [16,6]^−^ cage formed from (16,6) by Me^−^ abstraction.^69^ Bottom right: [16,6]^−^ cage formed from (16,7) by Me_2_Al^+^ cleavage.^48^ Me_3_Al end groups, characteristic for the anions, are indicated by the blue circle. Hydrogens are omitted for clarity. The energies and Gibbs free energies (*T* = 298 K, *p* = 1 atm) of the cage anions are given relative to the sheet anion. DG-c = estimate for condensed phase Gibbs free energy.

This new model, shown in Fig. 6, has a hexagonal Al 4-coordinate sheet structure, and it could form *via* aggregation of smaller sheet structures (see above). Comparison to its previously reported cage isomers, preferably forming from the most stable neutral (16,6) cage located by DFT calculations^60^ by Me^−^ abstraction,^69^ rather than from the higher energy (16,7) cage by Me_2_Al^+^ cleavage,^48^ is eye-opening: the sheet anion is as much as 66 kJ mol^−1^ lower in total energy, 94 kJ mol^−1^ lower in gas phase Gibbs free energy (*T* = 298 K, *p* = 1 atm), and 86 kJ mol^−1^ lower in Gibbs free energy after corrections for condensed phase^24,60,70–72^ (see computational details in ESI†). Each of the anions feature a Me_3_Al end group, as illustrated by the blue circles in Fig. 6.

The remarkable stabilization of the sheet anion in comparison to the cages arises from chelation of one of the methyl groups of Me_3_Al with the adjacent Me_2_Al end group, thus forming a six-membered ring in resemblance of bulk of the sheet.

It is also worth noting that the sheet anion features 24 potentially labile edge methyl groups, which is the number of low energy substitutions observed for methyl to ethyl exchanges in our previous alkyl scrambling study.^29^ With only 19 potentially labile methyl groups, the cage anions were in mismatch with those experiments, guiding us toward a more detailed investigation of alternative structural motifs.

As such, rearrangement would be required subsequent to an aggregation event, explaining the slow appearance of [16,6]^−^ following the rapid disappearance of the lower molecular weight species. Given the relatively low ion intensities observed even at the half hour mark compared to analyses of mature commercial samples, it is likely that only a fraction of the mixture has undergone *all* of the reactions (hydrolysis, aggregation, rearrangement) required for the formation of competent activators.

## Conclusions

While exceptional precautions are required to successfully study the growth of MAO oligomers mass spectrometrically (conditioning the instrument with a solution of TMA as a drying agent is a far from routine procedure), a considerable pay-off is obtained in the form of the only meaningful data thus far collected on this process. The ability to examine the dynamics of individual oligomers having undergone activation is a considerable advance in characterization capability, and the production of a solution dominated by the same ion ([16,6]^−^) observed in commercial samples is a remarkable observation considering the differences in reaction conditions between a small syringe and an industrial-scale reactor. The time course information suggests that the formation of higher oligomers does not involve incremental additions of Al_1_ species, and instead arises *via* aggregation of oligomers of intermediate size followed by rearrangement processes that decrease the overall Me : Al ratio. The approach and results described here are a revealing first step towards understanding and optimizing the formation of those components of MAO most capable of behaving as activators.

## Conflicts of interest

The work was partially funded with the support of NOVA Chemicals' Centre for Applied Research.

## Supplementary Material

SC-012-D0SC05075J-s001

SC-012-D0SC05075J-s002

SC-012-D0SC05075J-s003
