# Supplementary material for: Real-time analysis of methylalumoxane formation
Source: Chem Sci. 2020 Oct 27;12(2):546–51. doi: 10.1039/d0sc05075j (PMC8178985; doi:10.1039/d0sc05075j)
Supplement: SC-012-D0SC05075J-s003 [file SC-012-D0SC05075J-s003.pdf]

Electronic Supplementary Material (ESI) for Chemical Science.
This journal is © The Royal Society of Chemistry 2020

Supporting Information

Real-time analysis of methylalumoxane formation

Anuj Joshi,[a] Harmen S. Zijlstra,[a] Elena Liles,[a] Carina Concepcion,[a]
Mikko Linnolahti [b] and J. Scott McIndoe*[a]

a. *Department of Chemistry, University of Victoria, PO Box 1700 STN CSC, Victoria, BC V8W 2Y2, Canada.*
Fax: +1 (250) 721-7147; Tel: +1 (250) 721-7181; E-mail: mcindoe@uvic.ca

b. *Department of Chemistry, University of Eastern Finland, P.O. Box 111, FI-80101 Joensuu, Finland*

Experimental Section

Computational details

The structure of the [**16,6**]$^-$ anion was located by a systematic approach, which will be detailed in a separate publication.[1] The structure was optimized by Gaussian 16 software,[2] using the M06-2X metahybrid GGA functional of the Minnesota series[3] combined with the def-TZVP basis set by Ahlrichs *et al*.[4] The same methodology has been used in our recent computational investigations concerning MAO,[5] and it has been demonstrated to properly account for the dispersive interactions,[6] here arising from association of Me_3Al to the edges of the MAO sheet. Harmonic vibrational frequencies were calculated to confirm the structure as a true minimum in the potential energy surface, and to obtain gas-phase Gibbs free energy, which was calculated at T = 298K and p = 1atm. The condensed phase energy corrections to Gibbs free energy, were estimated by multiplication of the $T\Delta S$ term of Gibbs free energy by 2/3, as recommended and used in the previous literature.[7-11] For comparison, previously reported cage isomers[12-13] were added to the results (Table S1).

General Considerations

All experiments were performed under inert atmosphere using standard Schlenk and glovebox techniques. Fluorobenzene and 1,2-difluorobenzene was obtained from Oakwood Chemicals Ltd. and were refluxed over CaH_2, distilled under N_2, and stored over activated molecular sieves 4Å inside a glovebox for a minimum of 3 days prior to use. Me_3Al (2M in toluene) and OMTS (98 %) was purchased from Sigma-Aldrich and was used as received. Cp_2ZrMe_2 was purchased from Strem Chemicals.

All mass spectra were collected on a Micromass Q-ToF micro mass spectrometer in the positive or negative ion mode using electrospray ionization. Capillary voltage was set at 3000 V with source and desolvation gas temperature at 85 °C and 185 °C, respectively. The desolvation gas flow was set to 400 L h^{-1}. All MS/MS data was obtained as product ion spectra using 5.0 grade argon as the collision gas and a voltage range of 2–100 V. The ESI-MS spectra were recorded by injecting the solution from the glove box to the spectrometer via PTFE tubing (1/16” o.d., 0.005” i.d.) at a rate of 40 µL/min.

All experimental supplies, such as glass vial, syringes, gas-tight syringes, and PTFE tubings, were placed in a vacuum oven for 3 hours. Prior to the MS analysis, the materials were brought and stored inside a glovebox. Conditioning of the instrument was also done prior. A fresh capillary was used for each experiment to avoid unnecessary clogging issues. The baffle and sample cone were rinsed with dilute HCl/MeOH mixture between runs to ensure that they were free of Al_2O_3 film formation (Shown below).

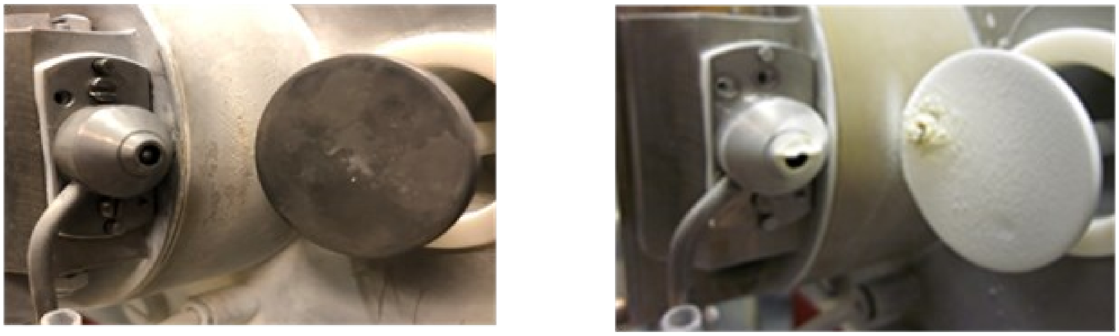

Figure S1. Baffle cone (left) before and (right) after running methylalumoxane.

Prior to analyzing these moisture and air sensitive samples, the PTFE tubing and attached instrument were conditioned by running a dilute solution (ca. 0.01 M) of Me_3Al. This precaution ensures the PTFE tubing and the MS are free of trace amounts of moisture and oxygen. The probe tip was also adjusted for these experiments so that it was close (approximately 1 mm) to the sample cone.

Solvent water estimation[14]

Difluorobenzene was distilled over CaH_2 and then collected. To the distilled solvent water was added. Using a separatory funnel, the organic layer was collected and then degassed using freeze pump thaw technique. The wet difluorobenzene was then taken to the glovebox and the water content in the solvent was then estimated using ^1H proton NMR.

Cp_2ZrMe_2 (18.4 mg) was collected in a 10 mL glass vial with 0.5 mL of difluorobenzene and 0.2 mL of dry benzene-d_6. A ^1H proton NMR was recorded and the amount of water in the solvent was calculated. The water concentration in the solvent was 0.055 M.

Monitoring experiments

A stainless-steel union tee was fitted with three lengths of PTFE tubing using PEEK ZDV low pressure nuts and ferrules. The two short, and equal lengths were fitted with Idex PEEK/PTFE syringe inlets using a PEEK union, and ZDV nut/ferrule. The entire apparatus was then dried in a vacuum oven before being transferred to a LC Technology Solutions LCB-120 glovebox. After flushing the entire apparatus with dry PhF via syringe, the measured, long length of PTFE tubing was connected to the QTOF Micro spectrometer. The dead time of this system was about 40 sec at a combined flow rate of 50 μL/min.

Syringe 1: 0.16 mL of 2 M Me_3Al was added to 5 mL of degassed difluorobenzene in a vial and quickly loaded into a 1 mL gas-tight syringe. *Syringe 2*: 16.8 mg of OMTS was dissolved in 5 mL of dry difluorobenzene. 0.4 mL of this solution was diluted with 10 mL difluorobenzene, degassed and quickly loaded into a 1 mL gas-tight syringe. The two syringes were inserted into a dual syringe pump with PTFE

tubing connected to the syringes. The tubing was connected to a mixing tee to combine the two solutions, and PTFE outlet tubing was attached to the mass spectrometer for analysis. The flow rates were set at 25 µL /min and ESI-MS data was collected continuously.

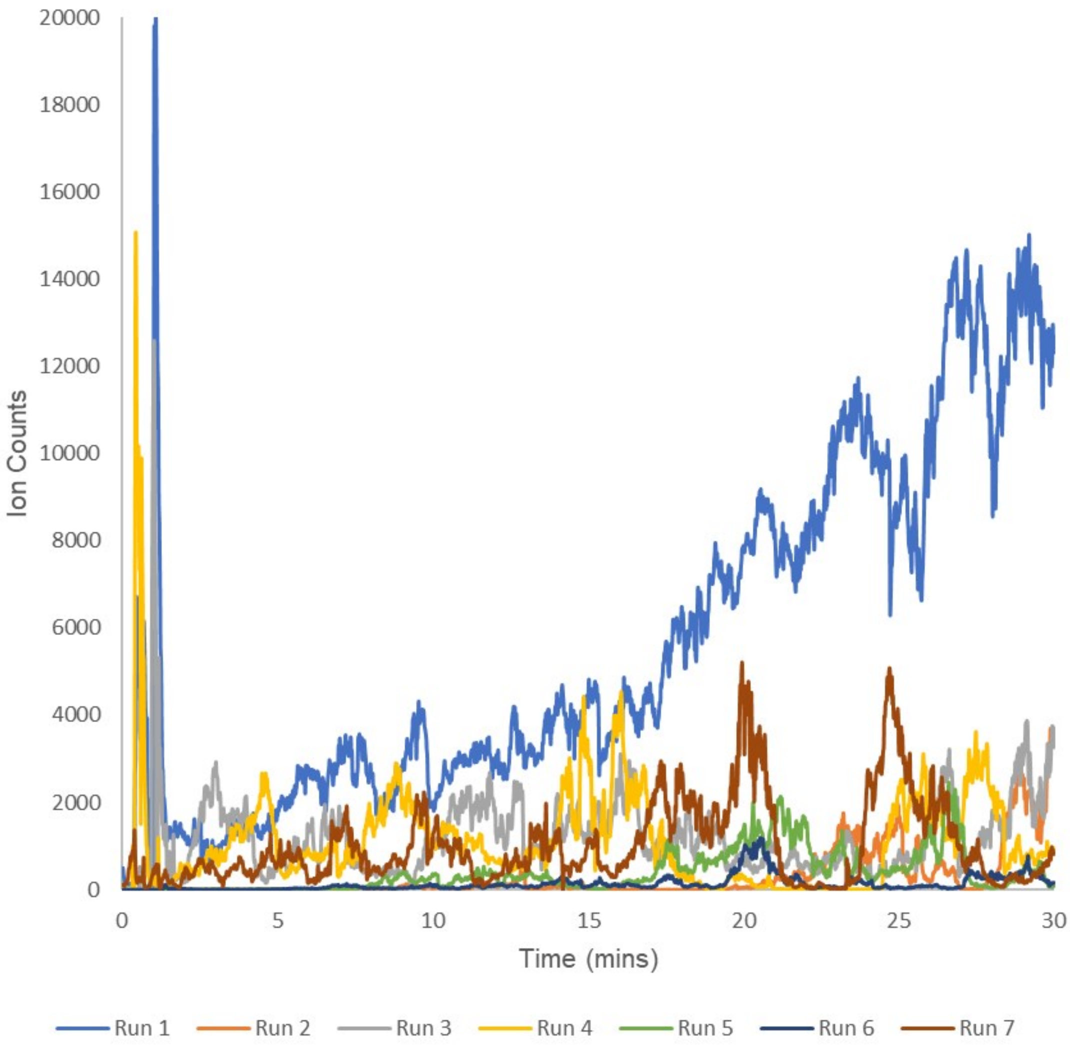

Figure S2. Total Ion Count (TIC) as a function of time for a typical ESI-MS run of MAO synthesis for 7 different experiments. The TIC was often highly errative as it is extremely challenging to run and analyze MAO mixtures. The most common problems are inconsistent spray/clogging while running these experiments. The error in the TIC are shown in Figure S3.

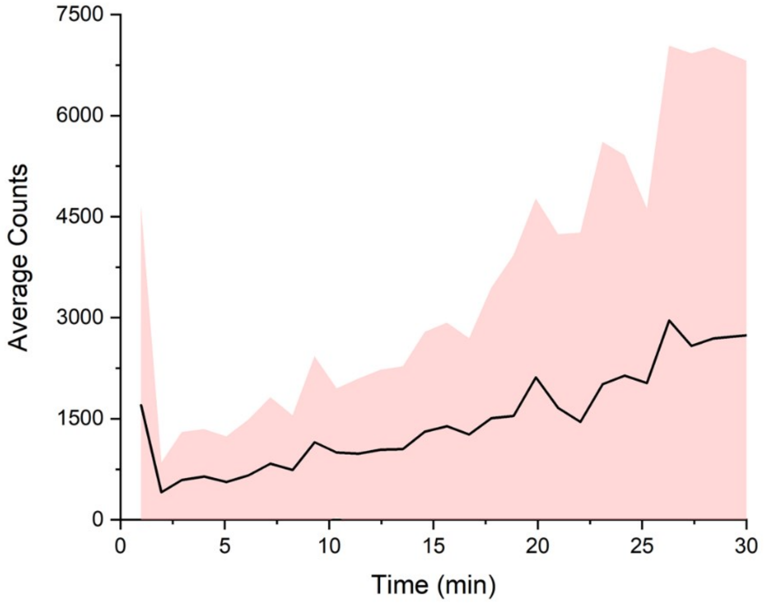

Figure S3. Error in the TIC for seven different experiments shown in red (standard deviation) and the average counts of all the experiments are shown by the black line.

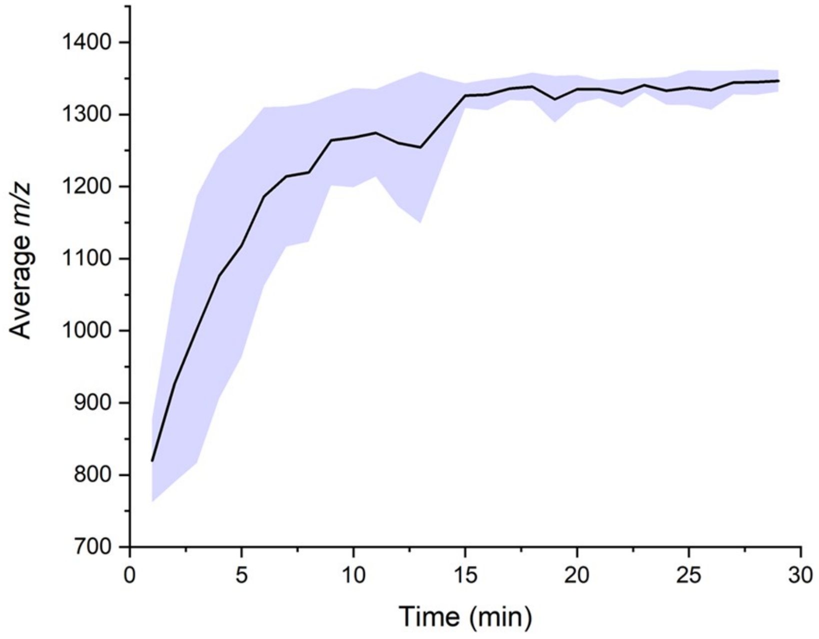

Figure S4. Error in the average molecular weight for MAO anions formed in different experiments in blue and the average *m/z* of all the experiments are shown by the black line.

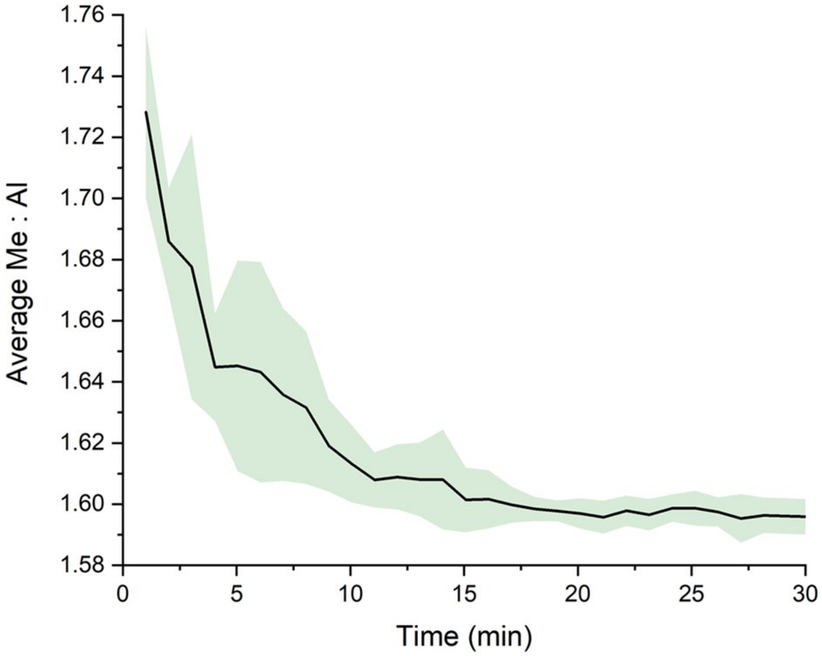

Figure S5. Error in the average Me:Al ratio for MAO anions formed in different experiments in green and the average Me:Al of all the experiments are shown by the black line.

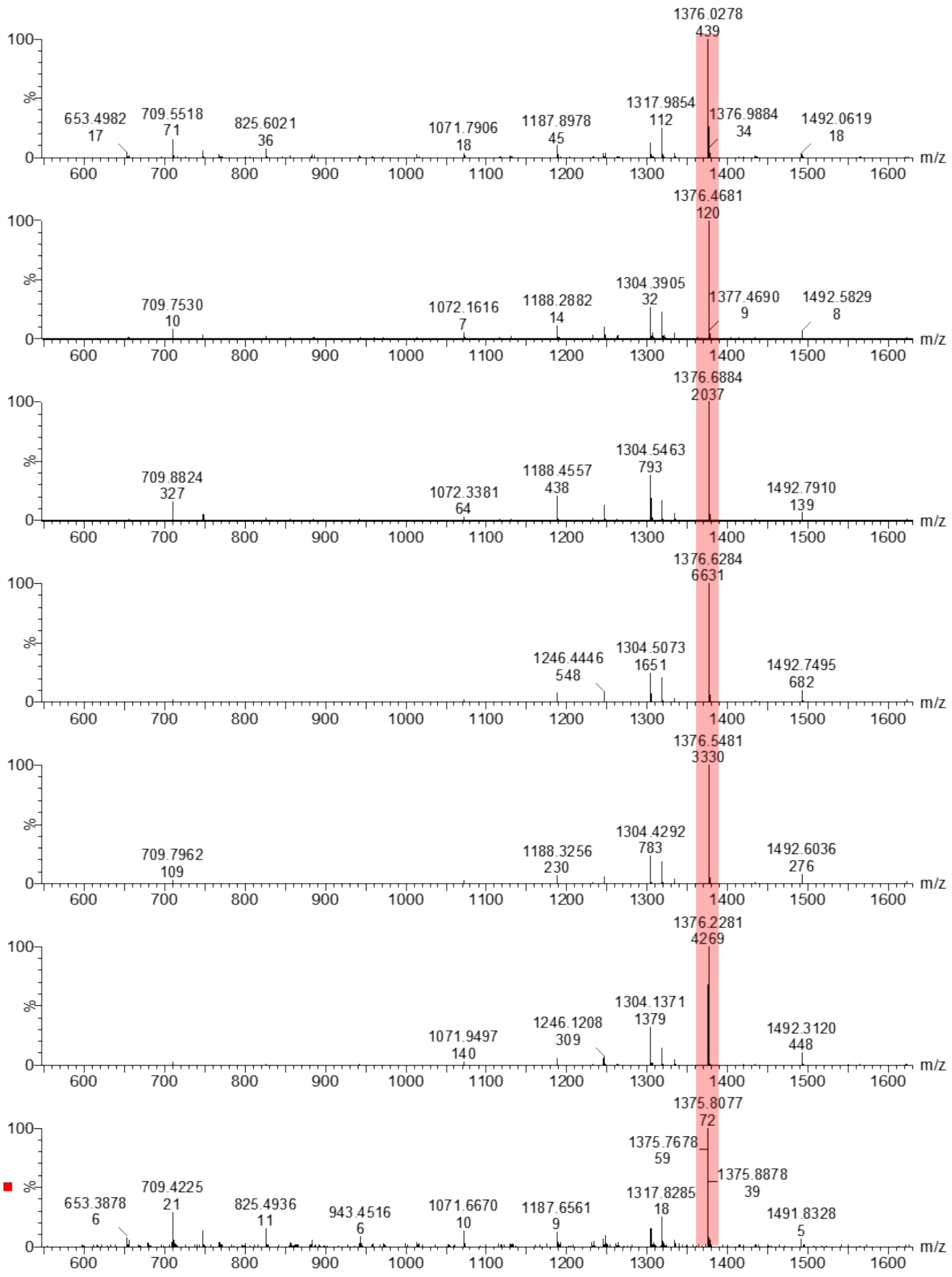

Figure S6. The anion distribution upon addition of TMA to wet (0.055 M H_2O), degassed difluorobenzene and OMTS after 10 mins for 7 different experiments. The results suggest that even though the TIC for different experiments is very different the speciation between different runs are similar. Red line shows the dominant [**16,6**]$^-$ anion which is also observed in commercial MAO samples. Anion distribution at different times for separate runs are shown in Figure S7-S13.

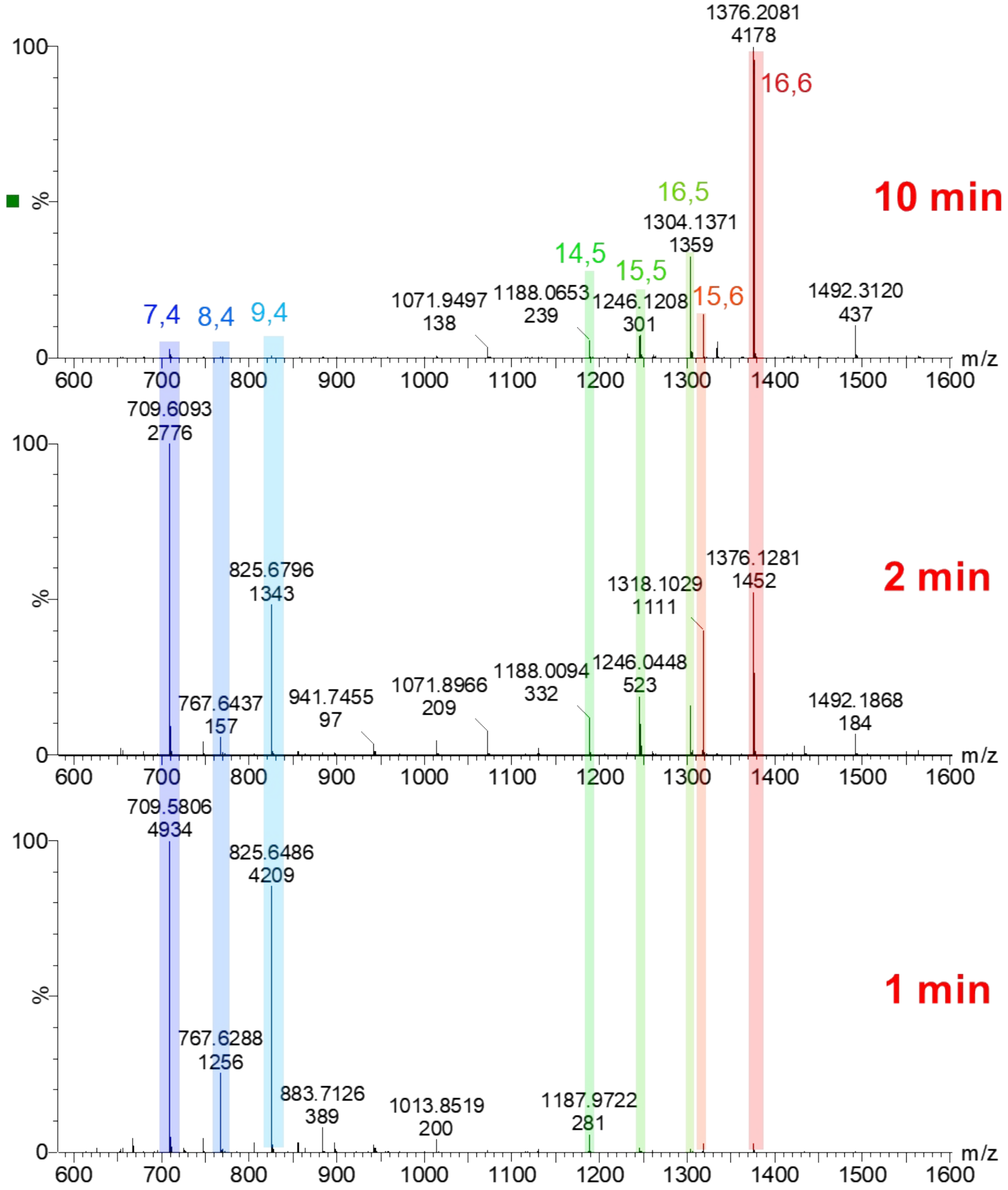

Figure S7. ESI-MS spectrum at 1, 2 and 10 minutes showing the change in the anion distribution during synthesis of MAO in wet (0.055 M H_2O), degassed difluorobenzene. Run #1 of 7.

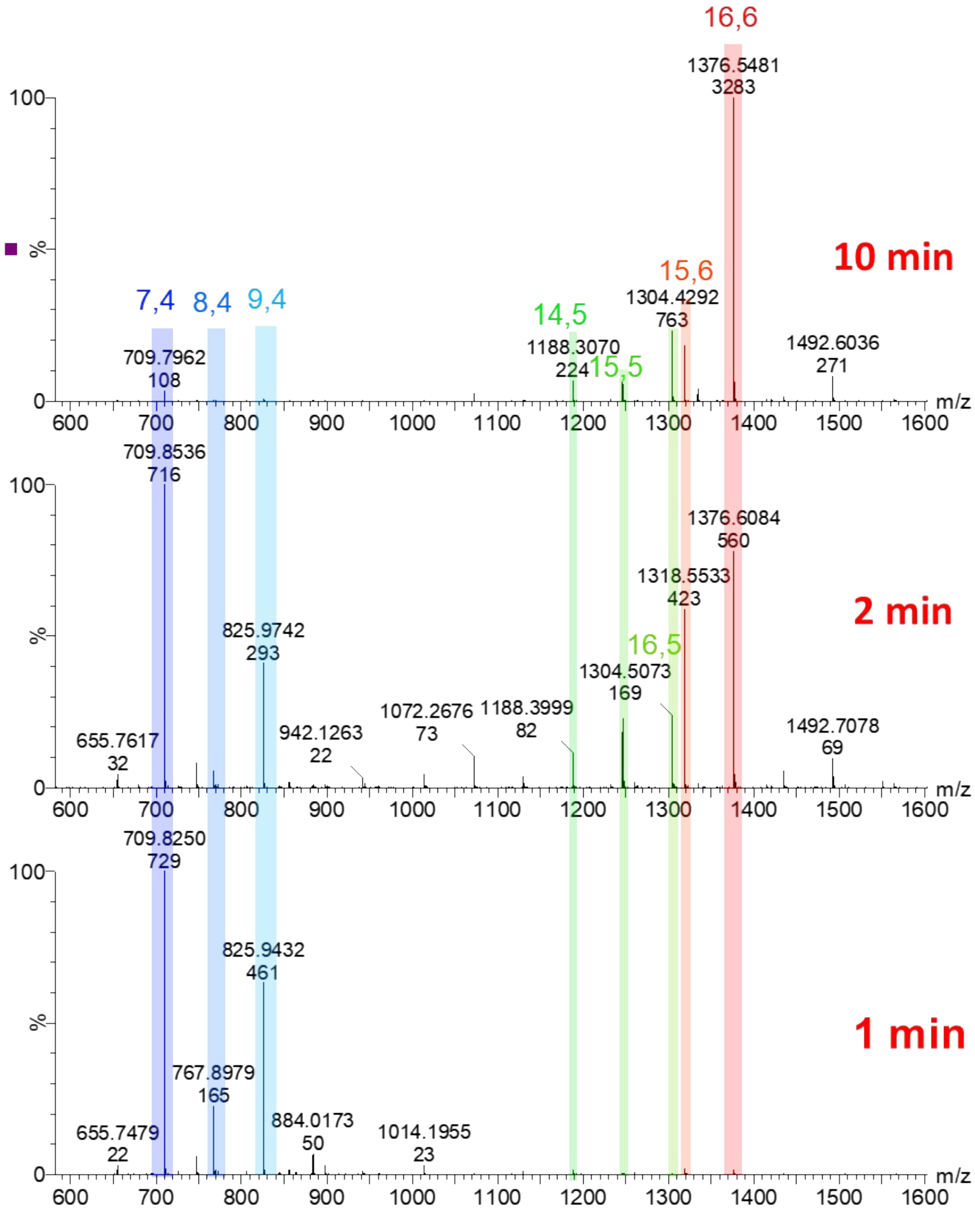

Figure S8. ESI-MS spectrum at 1, 2 and 10 minutes showing the change in the anion distribution during synthesis of MAO in wet (0.055 M H_2O), degassed difluorobenzene. Run #2 of 7.

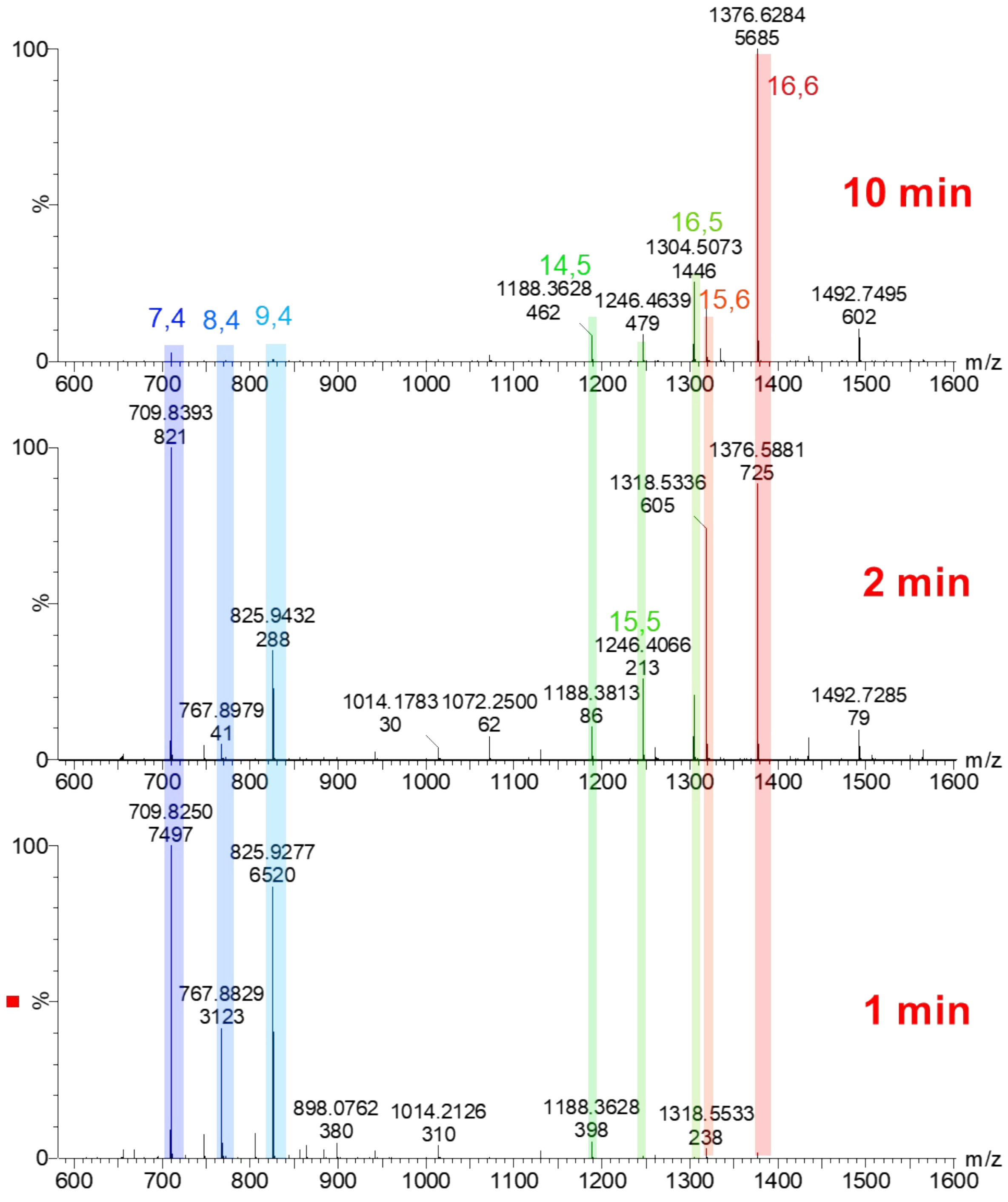

Figure S9. ESI-MS spectrum at 1, 2 and 10 minutes showing the change in the anion distribution during synthesis of MAO in wet (0.055 M H_2O), degassed difluorobenzene. Run #3 of 7.

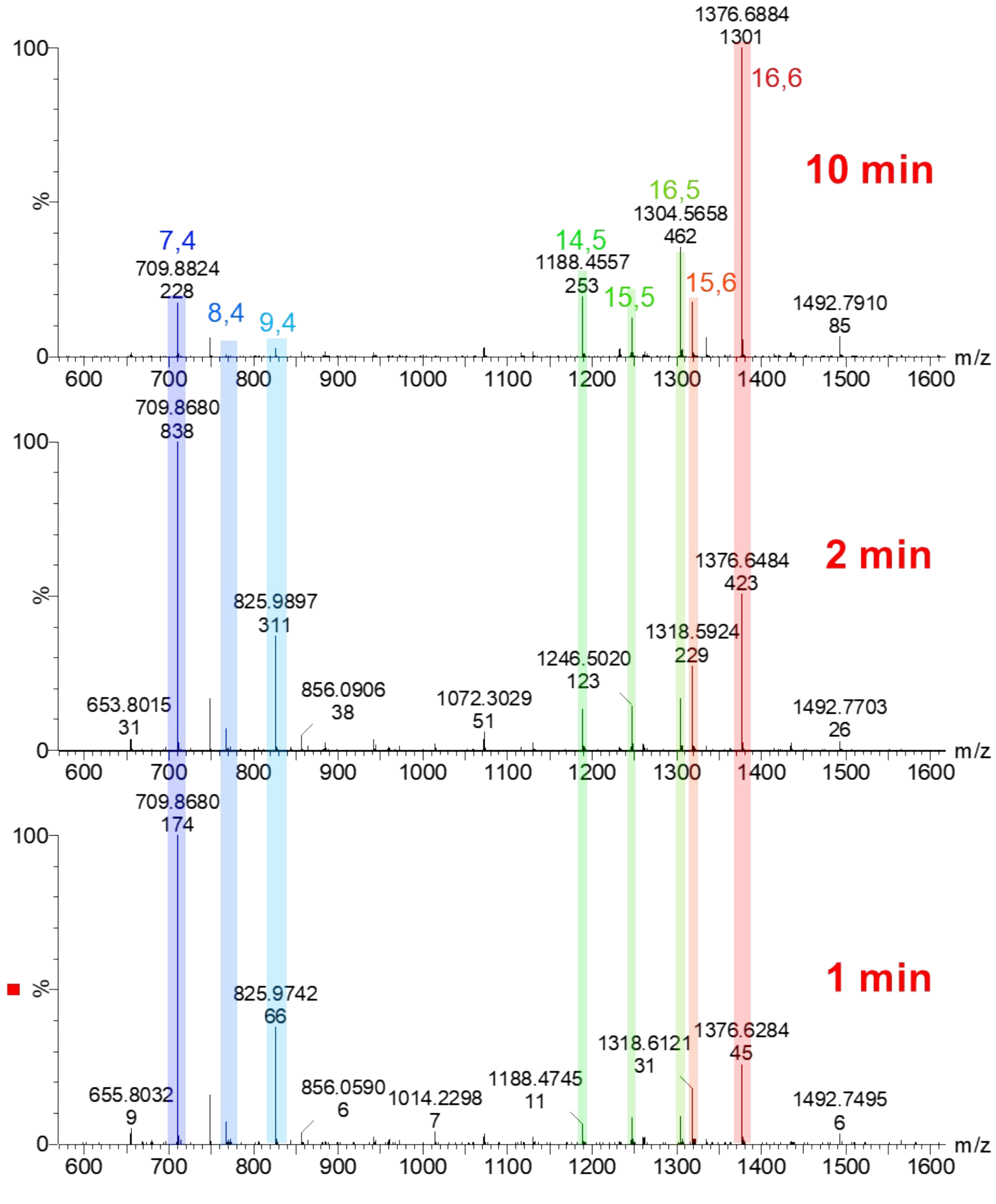

Figure S10. ESI-MS spectrum at 1, 2 and 10 minutes showing the change in the anion distribution during synthesis of MAO in wet (0.055 M H_2O), degassed difluorobenzene. Run #4 of 7.

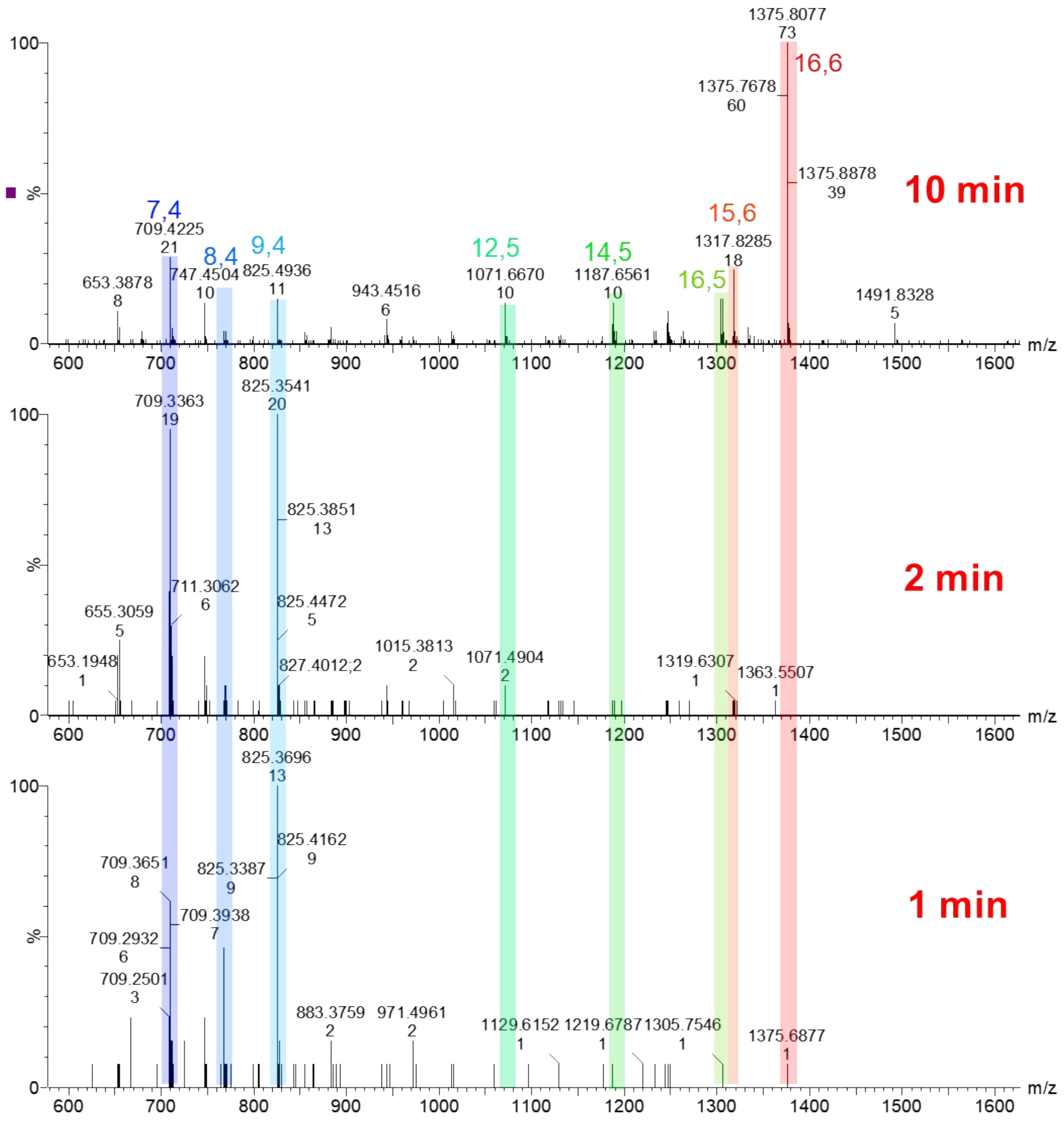

Figure S11. ESI-MS spectrum at 1, 2 and 10 minutes showing the change in the anion distribution during synthesis of MAO in wet (0.055 M H_2O), degassed difluorobenzene. Run #5 of 7.

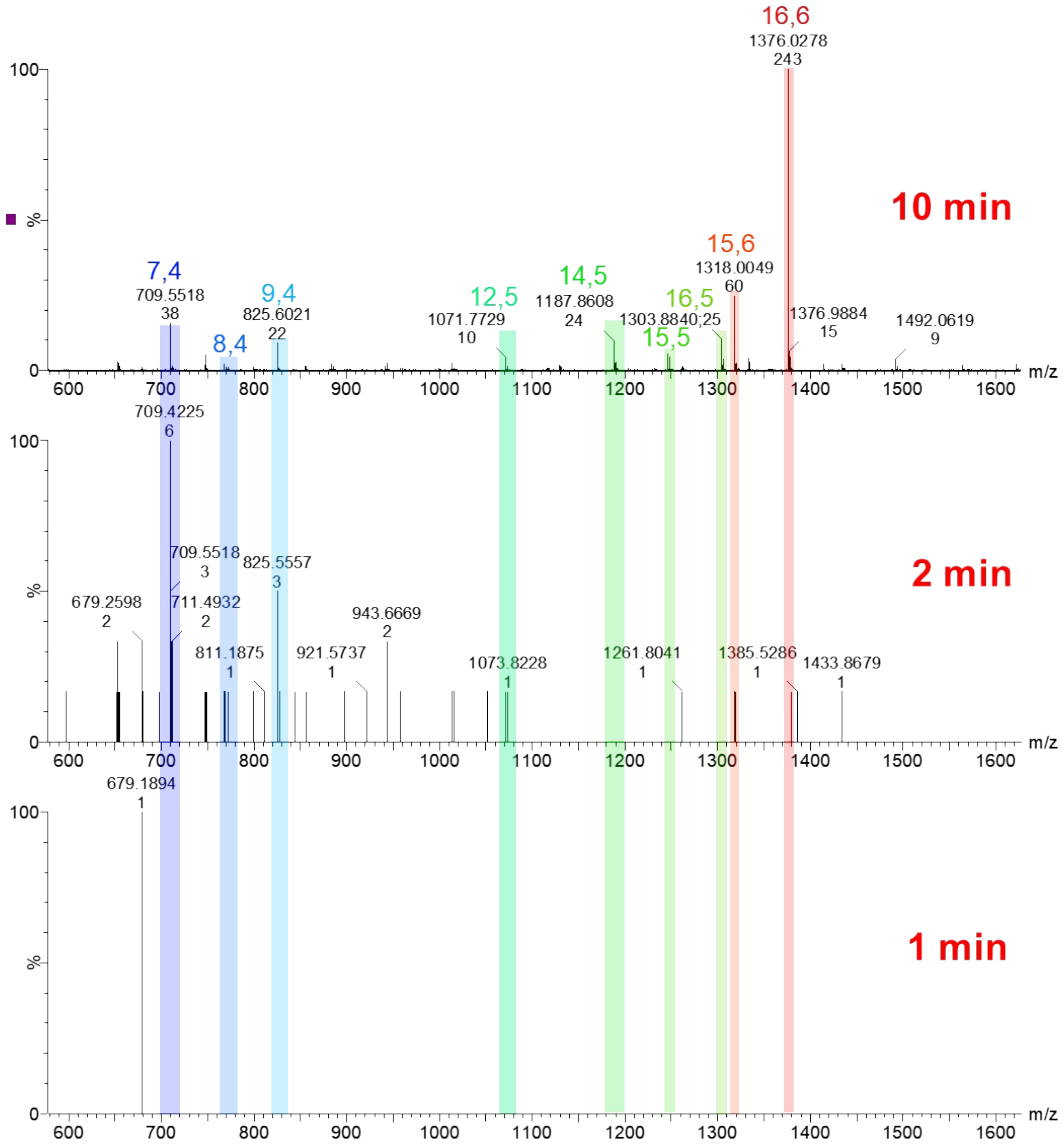

Figure S12. ESI-MS spectrum at 1, 2 and 10 minutes showing the change in the anion distribution during synthesis of MAO in wet (0.055 M H_2O), degassed difluorobenzene. Run #6 of 7.

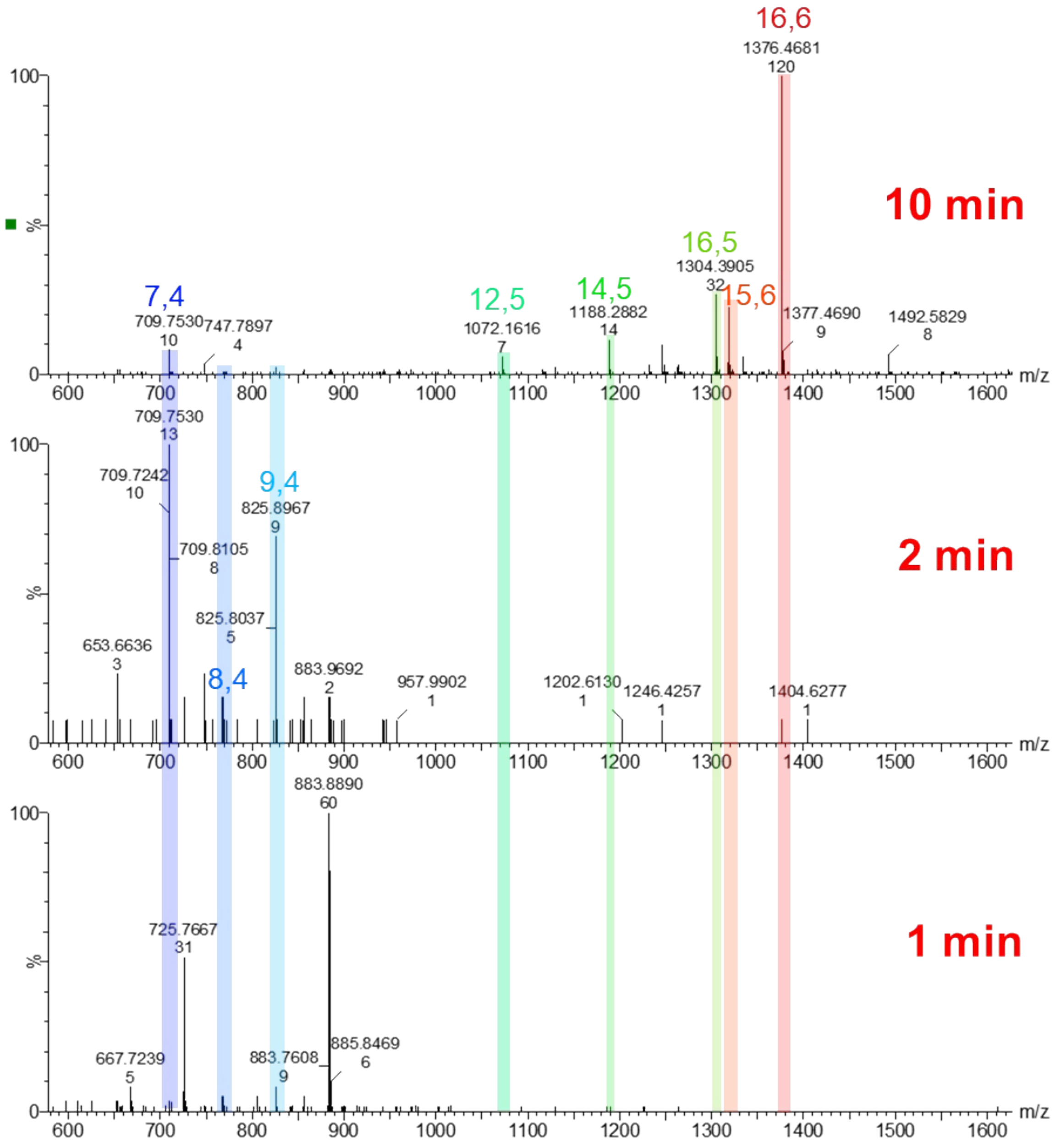

Figure S13. ESI-MS spectrum at 1, 2 and 10 minutes showing the change in the anion distribution during synthesis of MAO in wet (0.055 M H_2O), degassed difluorobenzene. Run #7 of 7.

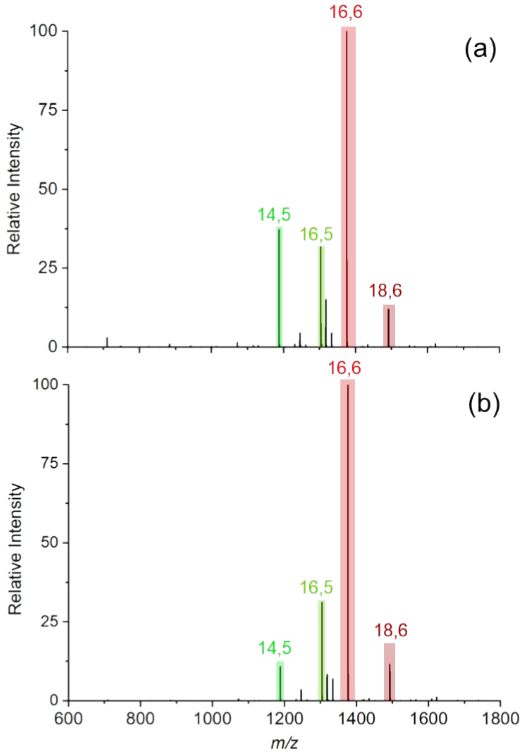

Figure S14. MS spectra after 20 mins of reaction of TMA with wet (0.055 M H_2O), degassed difluorobenzene when (a) additive (OMTS) is added from the start (offline) and (b) when OMTS is added through a mixing tee (on-line). These experiment shows that the addition of OMTS to generate the MAO anions could be done either at the start or during the analysis using a dual syringe pump.

The reaction of TMA and water was also studied in fluorobenzene, which has a much lower water content that does 1,2-difluorobenzene. Since the water content of PhF is 0.009 M the reaction is much slower compared to the reaction in difluorobenzene. The formation of [**16,6**]$^{-}$ happens over a span of 4 hours as compared to 10 mins in difluorobenzene. Heating the mixture after 4 hours in PhF leads to a spectrum dominated by [**16,6**]$^{-}$ (Figure S15).

We conducted a reaction of TMA and water in PhF at elevated temperatures i.e. 60 °C, and saw the formation of [**16,6**]$^{-}$ in 20 mins but not as a single dominant ion (Figure S16). The spectra recorded for analysis at higher temperature was also complicated as compared to room temperature due to oxidation of anions.

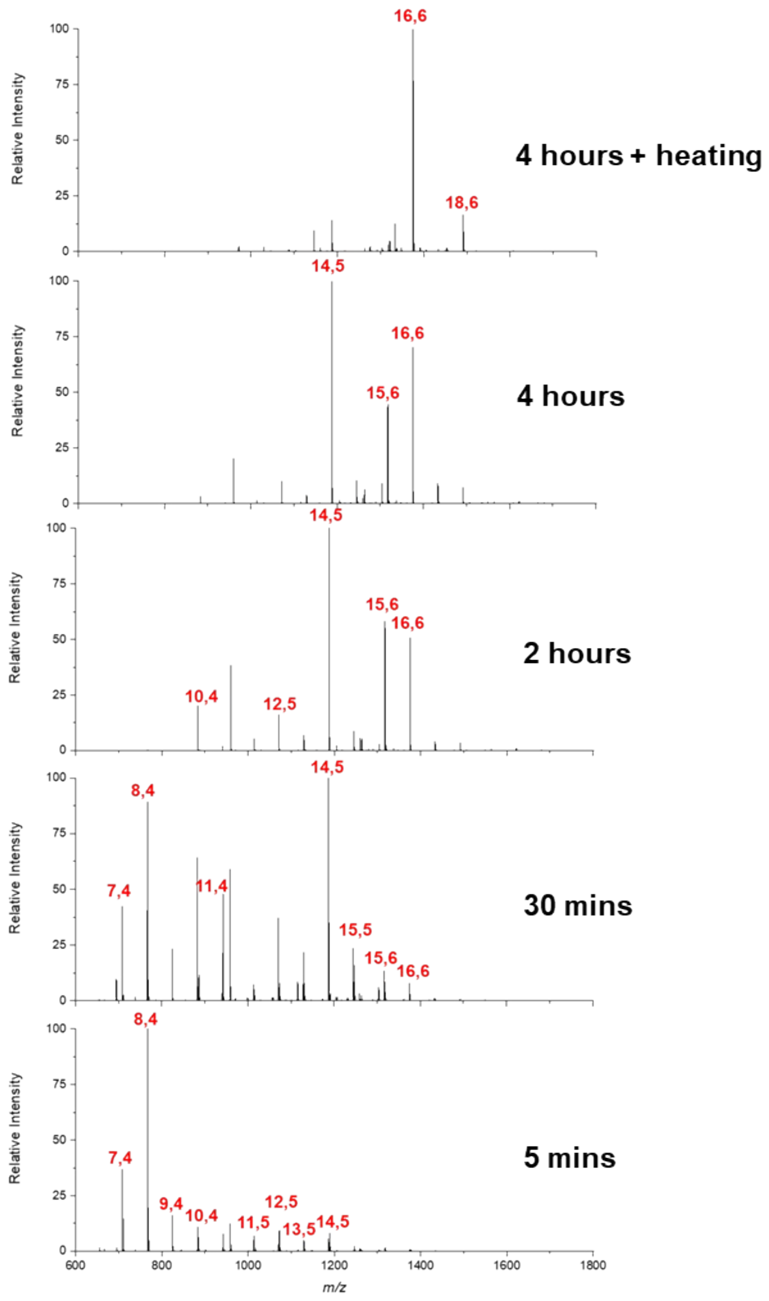

Figure S15. Reaction of TMA in PhF with [H_2O] = 0.009 M at 100:1 OMTS ratio. The lower amount of water slows down the reaction as compared to the reaction in difluorobenzene. The anion 16,6 is observed as a dominant anion upon heating the reaction mixture after 4 hours.

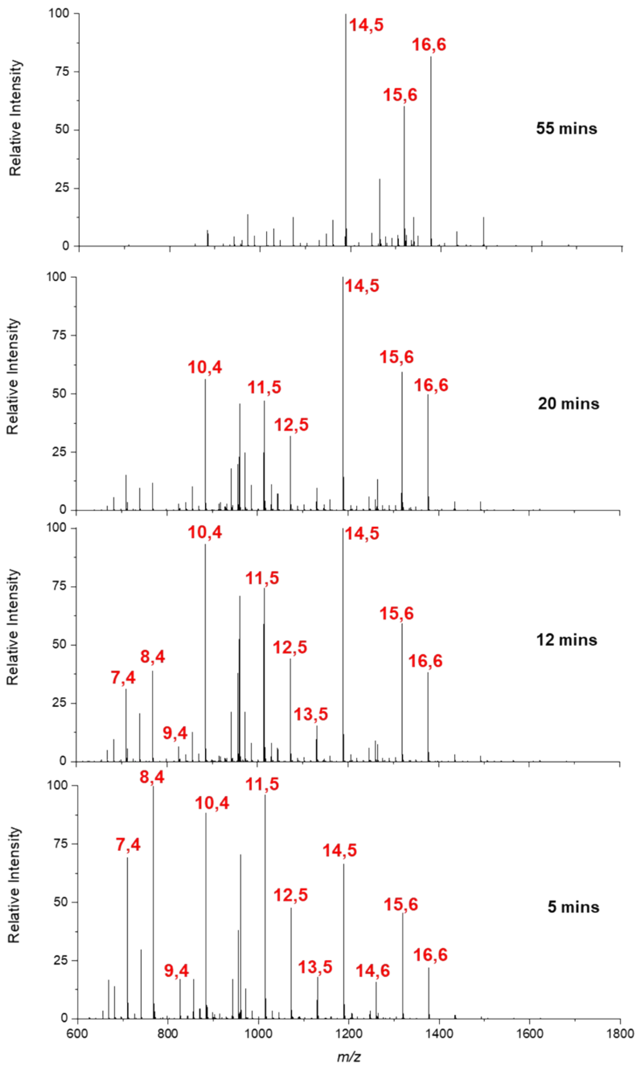

Figure S16. Reaction Monitoring at 60 degree in PhF with [H_2O] = 0.009 M at 100:1 OMTS ratio. The reaction is faster as compared to room temperature, but the spectrum is not as clean as the spectrum at room temperature. Also 16,6, is not observed as a single dominant anion under these conditions.

MS/MS Spectra

The MS/MS product ion spectra show fragmentation exclusively through loss of Me_3Al (-72 Da). The number of Me_3Al losses exceed the y values for a given ion because at high energies the ion can rearrange to generate free Me3Al via the process 3(MeAlO) → Al_2O_3 + Me_3Al.

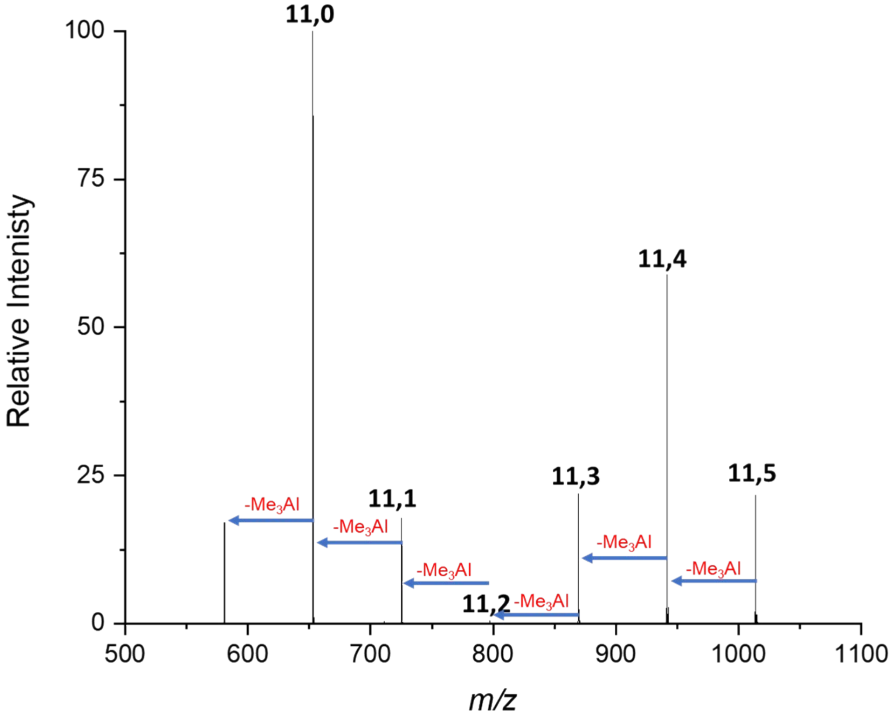

Figure S17. MS/MS spectrum of $[(MeAlO)_{11}(Me_3Al)_5Me]^-$.

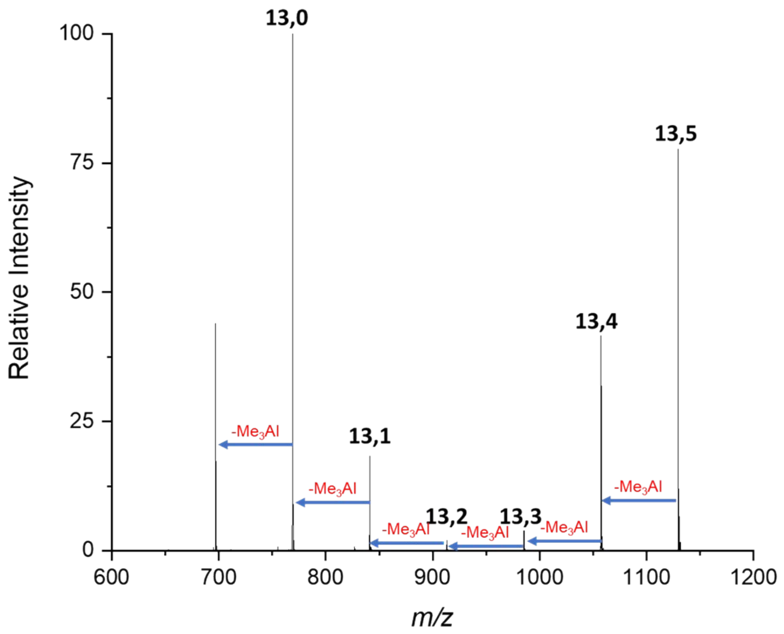

Figure S18. MS/MS spectrum of $[(MeAlO)_{13}(Me_3Al)_5Me]^-$.

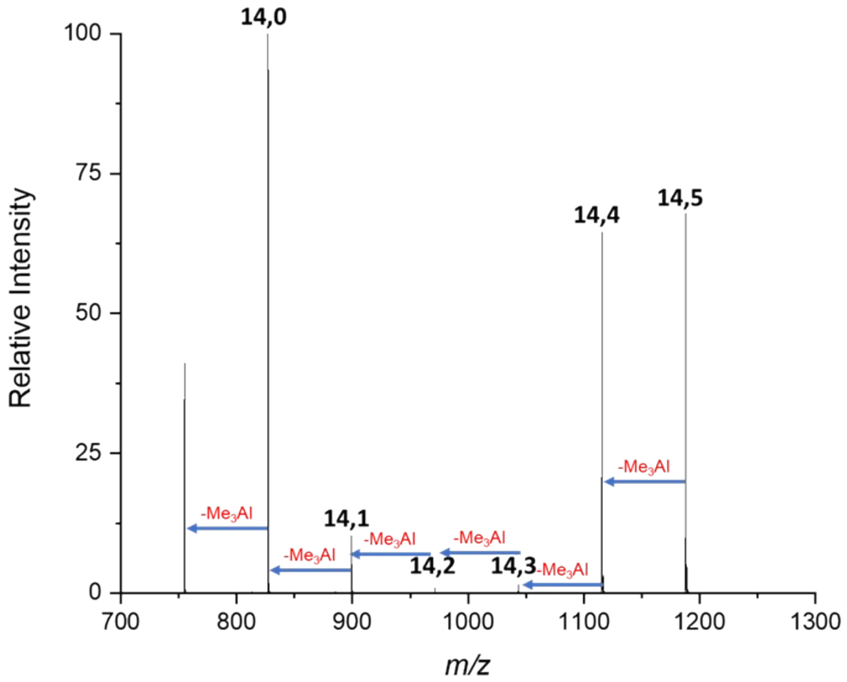

Figure S19. MS/MS spectrum of $[(MeAlO)_{14}(Me_3Al)_5Me]^-$.

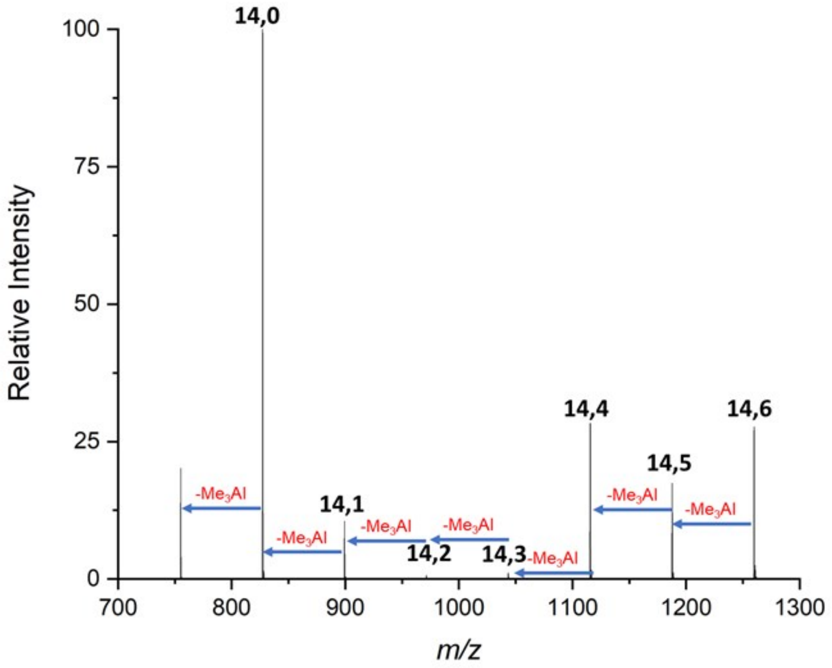

Figure S20. MS/MS spectrum of $[(MeAlO)_{14}(Me_3Al)_6Me]^-$.

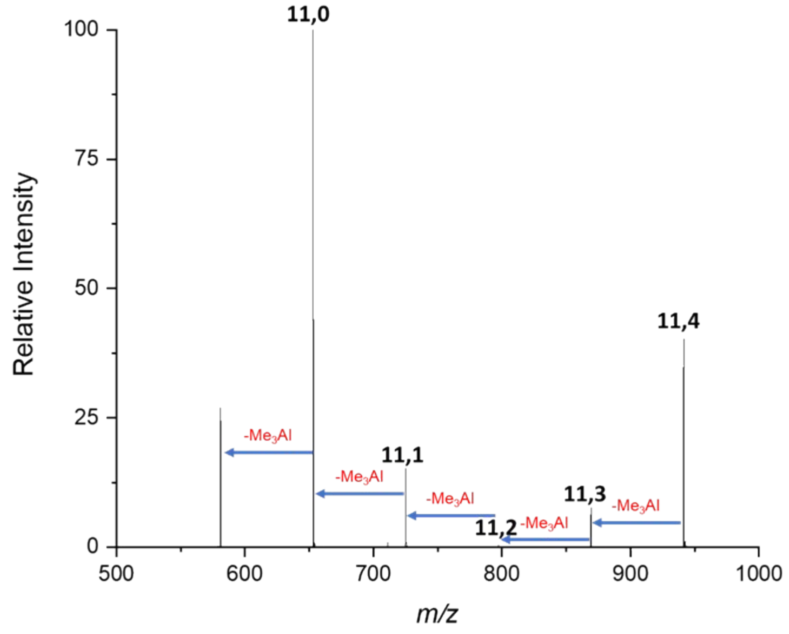

Figure S21. MS/MS spectrum of $[(MeAlO)_{11}(Me_3Al)_4Me]^-$.

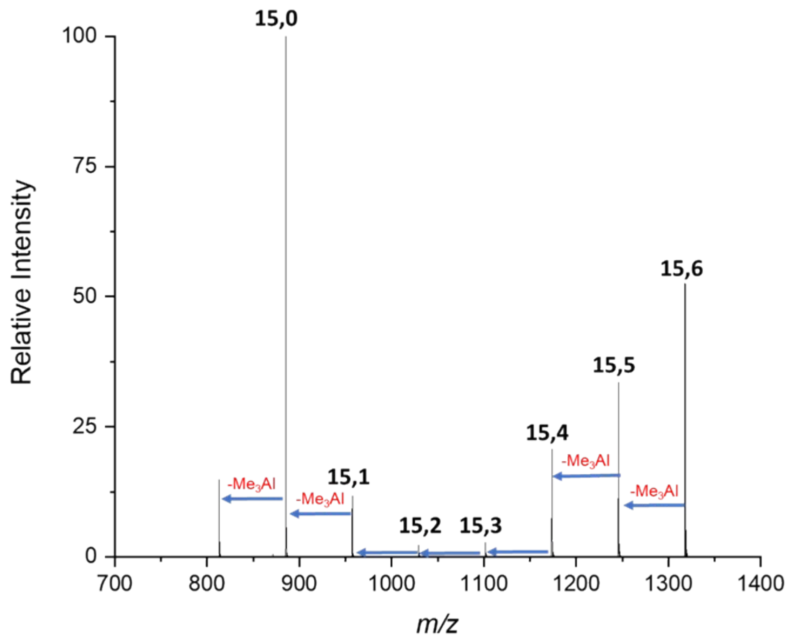

Figure S22. MS/MS spectrum of $[(MeAlO)_{15}(Me_3Al)_6Me]^-$.

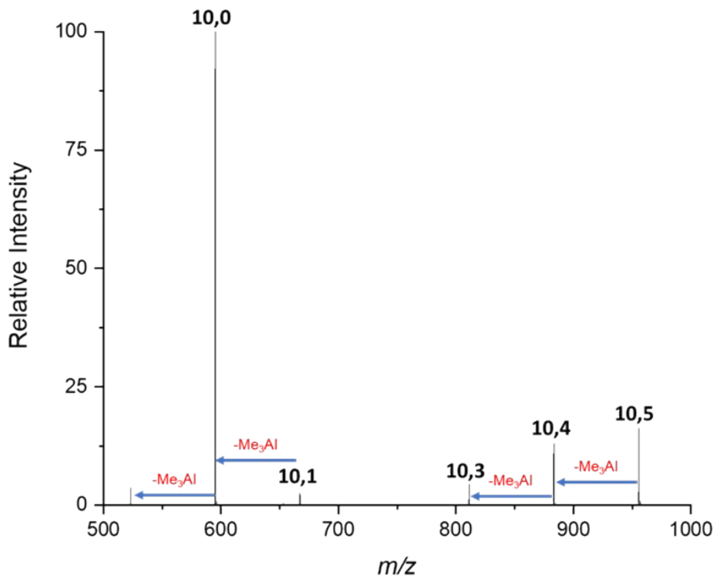

Figure S23. MS/MS spectrum of $[(MeAlO)_{10}(Me_3Al)_5Me]^-$.

Table S1. Calculated electronic energy, enthalpy, entropy and Gibbs energy of sheet and cage isomers of $[(MeAlO)_{16}(Me_3Al)_6Me]^-$

	E / a.u.	*H* / a.u.	*S* / cal $mol^{-1}K^{-1}$	*G* / a.u.
$[16,6]^-$ sheet (this work)	-7936.774519	-7935.279111	598.334	-7935.563398
$[16,6]^-$ cage formed from (16,6) by Me^- abstraction (ref. 52 of the main text)	-7936.749562	-7935.252878	578.056	-7935.527531
$[16,6]^-$ cage formed from (16,7) by Me_2Al^+ cleavage (ref. 52 of the main text	-7936.734443	-7935.239955	567.591	-7935.509636

References

[1] M. Linnolahti, *to be Published*.
[2] M. J. Frisch, G. W. Trucks, H. B. Schlegel, G. E. Scuseria, M. A. Robb, J. R. Cheeseman, G. Scalmani, V. Barone, B. Mennucci, G. A. Petersson, H. Nakatsuji, M. Caricato, X. Li, H. P. Hratchian, A. F. Izmaylov, J. Bloino, G. Zheng, J. L. Sonnenberg, M. Hada, M. Ehara, K. Toyota, R. Fukuda, J. Hasegawa, M. Ishida, T. Nakajima, Y. Honda, O. Kitao, H. Nakai, T. Vreven, J. A. Montgomery, Jr., J. E. Peralta, F. Ogliaro, M. Bearpark, J. J. Heyd, E. Brothers, K. N. Kudin, V. N. Staroverov, R. Kobayashi, J. Normand, K. Raghavachari, A. Rendell, J. C. Burant, S. S. Iyengar, J. Tomasi, M. Cossi, N. Rega, J. M. Millam, M. Klene, J. E. Knox, J. B. Cross, V. Bakken, C. Adamo, J. Jaramillo, R. Gomperts, R. E. Stratmann, O. Yazyev, A. J. Austin, R. Cammi, C. Pomelli, J. W. Ochterski, R. L. Martin, K. Morokuma, V. G. Zakrzewski, G. A. Voth, P. Salvador, J. J. Dannenberg, S. Dapprich, A. D. Daniels, O. Farkas, J. B. Foresman, J. V. Ortiz, J. Cioslowski, D. J. Fox., Gaussian 16, Revision B. 01. **2016**, Gaussian Inc, Wallingford CT. (46AD).
[3] Y. Zhao, D. G. Truhlar, Y. Zhao, · D G Truhlar, Theor Chem Acc. 2008, 120, 215–241.
[4] A. Schäfer, C. Huber, R. Ahlrichs, *J. Chem. Phys.* **1994**, *100*, 5829–5835.
[5] See e.g. M. Linnolahti, S. Collins, *ChemPhysChem* **2017**, *18*, 3369–3374.
[6] C. Ehm, G. Antinucci, P. H. M. Budzelaar, V. Busico, *J. Organomet. Chem.* **2014**, *772*, 161–171.
[7] S. Tobisch, T. Ziegler, *J. Am. Chem. Soc.* **2004**, *126*, 9059–9071.
[8] C. Ehm, R. Cipullo, P.H.M. Budzelaar, V. Busico, *Dalton Trans.* **2016**, *45*, 6847–6855.
[9] M. Linnolahti, S. Collins, *ChemPhysChem* **2017**, *18*, 3369–3374.
[10] F. Zaccaria, R. Cipullo, P.H.M. Budzelaar, V. Busico, C. Ehm, *J. Polym. Sci. Part A Polym. Chem.* **2017**, *55*, 2807–2814.
[11] F. Zaccaria, C. Ehm, P.H.M. Budzelaar, V. Busico, *ACS Catal.* **2017**, 1512–1519.
[12] H. S. Zijlstra, M. Linnolahti, S. Collins and J. S. McIndoe, *Organometallics*, 2017, **36**, 1803–1809
[13] T. K. Trefz, M. A. Henderson, M. Linnolahti, S. Collins and J. S. McIndoe, *Chem. – A Eur. J.*, 2015, **21**, 2980–2991.
[14] N. A. Yakelis, R. G. Bergman, *Organometallics* **2005**, *24*, 3579–3581.
